# Urinary Malondialdehyde Is Associated with Visceral Abdominal Obesity in Middle-Aged Men

**DOI:** 10.1155/2015/524291

**Published:** 2015-10-11

**Authors:** Sun Min Lee, Young Hye Cho, Sang Yeoup Lee, Dong Wook Jeong, A Ra Cho, Jeong Suk Jeon, Eun-Ju Park, Yun Jin Kim, Jeong Gyu Lee, Yu Hyeon Yi, Young Jin Tak, Hye Rim Hwang, Seung-Hun Lee, Junehee Han

**Affiliations:** ^1^Department of Laboratory Medicine and Molecular Genetics and Research Institute of Convergence of Biomedical Science and Technology, Pusan National University Yangsan Hospital, Yangsan 626-770, Republic of Korea; ^2^Family Medicine Clinic and Research Institute of Convergence of Biomedical Science and Technology, Pusan National University Yangsan Hospital, Yangsan 626-770, Republic of Korea; ^3^Department of Family Medicine, Pusan National University Hospital, Busan 49241, Republic of Korea; ^4^Research And Statistical Support, Research Institute of Convergence for Biomedical Science and Technology, Pusan National University Yangsan Hospital, Yangsan 626-770, Republic of Korea

## Abstract

The purpose of the present study was to investigate multiple anthropometric parameters used to evaluate obesity, particularly visceral abdominal fat area, and various metabolic parameters including malondialdehyde (MDA) as an oxidative stress marker. We evaluated various measures of obesity, including body mass index (BMI), waist circumference (WC), sagittal abdominal diameter, fat percentages using dual-energy X-ray absorptiometry, visceral fat area (VFA), subcutaneous fat area, multiple biomarkers related to metabolic disease, and urinary MDA, in 73 asymptomatic middle-aged men who were not severely obese. We examined relationships between multiple measures of obesity, metabolic markers, and urinary MDA levels and evaluated associations between VFA and urinary MDA. In the visceral obesity group, *γ*-glutamyl transferase (GGT), uric acid, and urinary MDA levels were significantly higher than in the nonvisceral obesity group (*P* = 0.008, *P* = 0.002, and *P* = 0.018). Urinary MDA (*r* = 0.357, *P* = 0.002) and uric acid (*r* = 0.263, *P* = 0.027) levels were only significantly positively correlated with VFA among measures of obesity. Urinary MDA, serum GGT, and serum CRP were significantly positively associated with VFA (*P* = 0.001, *P* = 0.046, and *P* = 0.023, resp.), even after adjusting for BMI and WC.

## 1. Introduction

Obesity is associated with hypertension, dyslipidemia, metabolic syndrome, and type 2 diabetes [1, 2]. In particular, visceral abdominal fat accumulation is closely linked to insulin resistance and cardiovascular disease (CVD) [3, 4]. Obesity is usually defined by body mass index (BMI), waist circumference (WC), or body fat percentage; however, these measures are limited in their ability to distinguish visceral obesity, which plays a key role in the pathogenesis of cardiometabolic syndrome. Although their average BMI is low, Asians demonstrate higher fat percentages compared with Caucasians of equivalent BMI and have higher prevalence of diabetes and CVD [5, 6]. Alanine aminotransferase (ALT) [7], *γ*-glutamyl transferase (GGT) [8], uric acid [9], and C-reactive protein (CRP) [10] are suggested to be metabolic markers associated with hepatic dysfunction, oxidative stress, and inflammation.

Oxidative stress refers to an imbalance between oxidants and antioxidants on a cellular or individual level [11]. Many studies found higher oxidative stress levels in obese than nonobese people [12–14], which may be due to several potential mechanisms including chronic inflammation [15–17], hyperglycemia [18], or impairment of antioxidant defense systems [19, 20]. Malondialdehyde (MDA) has been recognized as an important indicator of lipid peroxidation that is generated as an end product from oxidative degradation of polyunsaturated fatty acids [21, 22]. Urinary MDA is especially useful as a noninvasive test for monitoring oxidative stress.

Studies that evaluate the correlation between various measures of obesity and metabolic parameters, focusing on visceral obesity, are scarce. Therefore, the present study was designed to investigate the associations between multiple anthropometric parameters of obesity, particularly those relating to visceral abdominal fat area, and various metabolic parameters in middle-aged men who were not severely obese. We were particularly interested in the association between visceral obesity and metabolic parameters, especially urinary MDA, after adjusting for BMI and WC.

## 2. Material and Methods

### 2.1. Study Subjects and Ethics

The study was approved by the Institutional Review Board at Pusan National University Yangsan Hospital, and informed written consent was obtained from all subjects before participating. Study subjects included 80 asymptomatic men between the ages of 40 and 60 years who visited the health promotion center in Pusan National University Yangsan Hospital. Subjects with a history of malignancy or a cardiovascular event and subjects receiving medication for acute diseases, such as myocardial infarction and angina pectoris, were excluded. Subjects with low body weight (BMI < 18.5 kg/m^2^) or severe obesity (BMI ≥ 30.0 kg/m^2^) were also excluded. Seventy-three middle-aged men were enrolled in this study.

### 2.2. Measurements

Following an overnight fast, blood samples were obtained from an antecubital vein between 08:00 and 09:00 a.m. ALT, GGT, and uric acid concentrations were measured using the Hitachi 7600 Analyzer (Hitachi Co., Ltd., Tokyo, Japan) by an enzymatic colorimetric method. Low-density lipoprotein and high-density lipoprotein were measured with Toshiba TBA200FR using a direct measurement method and triglycerides (TG) were measured using lipase, GK, GPO, and POD with a glycerol blank. CRP was measured using a Behring BN II nephelometer (Dade Behring, Marburg, Germany).

Height and weight were measured down to the first decimal place, and BMI was calculated as body weight (kg) divided by the squared height (m). Using a tape measure, WC was measured from the half-way point between the lower line of the last rib and the upper line of the iliac crest when a subject exhaled and was measured down to the first decimal place. Each subject's sagittal abdominal diameter (SAD) was measured in a supine position with legs extended using a portable sliding-beam caliper (Holtain Ltd., Dyfed, Wales, UK). The vertical abdominal length was measured in a supine position by letting the subject slightly lift the midsection up and inserting the fixed lower arm of the portable sliding-beam caliper at the location of iliac crest of pelvis (space between L4 and L5), letting the subject inhale deeply and slowly exhale, and lowering the upper arm of the portable sliding-beam caliper down until it touched the abdomen without pressing it. Measurements were taken to the nearest tenth of a cm [23].

Abdominal fat was assessed from computed tomography (CT) scans taken at the L4-L5 level. Abdominal fat was defined as the area corresponding to the pixel range from −190 to −30 Hounsfield units [24]. The visceral and subcutaneous abdominal adipose tissue areas were measured. The fat inside the peritoneum was considered visceral adipose tissue, and the fat between the dermis and muscle fascia was considered subcutaneous adipose tissue. Whole body fat composition was obtained using dual-energy X-ray absorptiometry (DXA) (Hologic Inc., Bedford, MA, USA).

Data on alcohol intake and smoking habits were obtained by interview. Subjects were divided into two groups by the amount of alcohol consumption: nondrinker 0–180 g/week and drinker >180 g/week. Smoking status was classified as nonsmoker or (former or current) smoker. Both diet and physical activity were assessed due to their possible effects on insulin sensitivity. Diet was monitored by using a semiquantitative food frequency questionnaire [25] and physical activity was assessed using the International Physical Activity Questionnaire [26]. Physical activity levels are expressed in MET-minute.

### 2.3. Urine MDA

Urinary MDA was measured with high performance liquid chromatography (HPLC). For the quantification of urinary MDA by HPLC [27], 3.0 mL (1%) phosphoric (V) acid, 0.4 mL ultrapure water, and 0.6 mL of sample or standard (1–125 *μ*M MDA) were added to a screw-capped test tube and mixed thoroughly. Then, 1.0 mL of 0.67% thiobarbituric acid was added to all tubes and kept in a 95°C water bath for 1 hour. After incubation, the tubes were placed in an ice bath. Then, the tubes were centrifuged for 15 minutes. The supernatant was filtered before being applied to a Zorbax Eclipse XDB-C8 (I.d. 4.6 × 250 mm, 5 *μ*m, Agilent). Measurements were made with a UV-Vis detector (Agilent 1100 series HPLC system, USA) at 532 nm. The mobile phase was 0.05 M potassium phosphate buffer (pH 6.8) with methanol (50 : 50, v/v). The flow rate was 0.5 mL/min. MDA and creatinine were analyzed in two voided specimens. Urinary MDA levels were expressed as *μ*mol/g creatinine, averaged, and used for analysis.

### 2.4. Statistical Analysis

Descriptive data were expressed as the mean value (±SD) or number (%). The 73 subjects were divided into two groups: the visceral obesity (VO) group and nonvisceral obesity (non-VO) group. The visceral obesity group consisted of the subjects whose CT visceral fat area (CT-VFA) was ≥100 cm^2^. The Shapiro-Wilk test was used to test the normality of the variables. To compare the means of two groups, we used the two-sample* t*-test or the Mann-Whitney *U* test for continuous variables depending on the normality of the variables. For the categorical variables, the Chi-square test was used to verify the group effect. Correlations between variables were tested using Spearman's correlation coefficients. Finally, multiple regression analysis was performed to investigate the relationship among the variables considering GGT, UA, CRP, TG, and urinary MDA levels as dependent variables and abdominal VFA as the independent variable after adjusting for BMI and WC. The analysis was conducted using SPSS version 18.0 for Windows (SPSS Inc., Chicago, IL). Statistical significance was accepted for *P* values <0.05.

## 3. Results

### 3.1. Clinical Characteristics of Subjects

The general characteristics of the study subjects are presented in Table 1. The overall average age was 51.2 years and mean BMI was 24.4 kg/m^2^. Mean abdominal VFA was 100.8 cm^2^.

### 3.2. Differences between the Characteristics of Patients in the Two Study Groups

No differences in age, smoking status, alcohol habits, physical activity, or dietary intake were observed between the VO group and non-VO group (Table 2).

The VO group had significantly higher BMI, WC, and SAD than the non-VO group (*P* = 0.009, *P* = 0.002, and *P* = 0.003, resp.), and DXA-measured trunk fat and total fat percentages were also significantly higher in the VO group (*P* = 0.005 and *P* = 0.014, resp.). However, there were no differences in DXA-measured upper arm fat and lower leg fat percentages. CT-measured subcutaneous fat area was similar between the two groups (*P* = 0.117). GGT and uric acid levels were higher in the VO group (*P* = 0.008 and *P* = 0.002, resp.), and TG levels were marginally higher in the VO group (*P* = 0.063). Urinary MDA levels were significantly higher in the VO group (*P* = 0.018).

### 3.3. Correlations between Obesity Measures, Metabolic Parameters, and Urinary MDA

BMI, WC, and DXA-measured total fat percentages were significantly positively correlated with ALT, GGT, CRP, and TG (Table 3), but not significantly correlated with uric acid or urinary MDA. However, urinary MDA (*r* = 0.357, *P* = 0.002) and uric acid (*r* = 0.263, *P* = 0.027) levels were only significantly positively correlated with VFA among the measures of obesity.

### 3.4. Multiple Regression Analysis of Abdominal VFA and Metabolic Parameter and Urinary MDA

In all subjects, the association between VFA and GGT (*β* = 0.23, *P* = 0.028) and CRP (*β* = 0.278, *P* = 0.01), as well as urinary MDA (*β* = 0.362, *P* = 0.001), remained significant after adjusting for BMI (Table 4). These associations also remained after adjusting for BMI and WC (GGT: *β* = 0.207, *P* = 0.046; CRP: *β* = 0.245, *P* = 0.023; urinary MDA: *β* = 0.349, *P* < 0.001). On the other hand, the associations between VFA and uric acid or TG were not significant after adjusting for BMI and WC.

## 4. Discussion

In the present study, we investigated associations between abdominal VFA and common clinical metabolic biomarkers in middle-aged men without morbid obesity. In addition, we were particularly interested in the correlation between urinary MDA, a known oxidative stress marker, and abdominal visceral adiposity. We demonstrated that urinary MDA, GGT, and CRP were significantly positively associated with VFA, even after adjusting for BMI and WC.

Oxidative stress is considered a crucial factor because this is an early instigator of metabolic syndrome [12] and a contributor to the development of major obesity-related comorbidities such as CVD [13]. MDA is a biomarker derived from lipid peroxides and that is considered useful marker of oxidative marker [28]. Previously published data showed that participants with a high VFA (≥100 cm^2^) were more likely to have high plasma MDA levels, which is consistent with our findings, although our study examined urine MDA level [29]. We checked urinary MDA as oxidative stress marker because that is noninvasive test. In our data, urine MDA is a marker of oxidative stress in obese people, especially those with visceral obesity.

We wondered if previously known cardiometabolic biomarkers as well as urinary MDA levels have been connected with various measures of obesity and specifically those laboratory markers that reflect visceral obesity. We confirmed that urinary MDA levels were related to abdominal visceral fat area after adjusting for BMI and WC, which are typically used to evaluate clinical obesity. Urinary MDA levels can indicate inflamed adipose tissue. Further study is required to determine the clinical utility of urinary MDA levels.

Serum uric acid was higher in the VO group than the non-VO group and showed a positive correlation with VFA; however, after adjusting for BMI and WC, there was no association with VFA. On the other hand, GGT and CRP demonstrated a significant association with VFA even after adjusting for BMI and WC. Previous studies showed GGT was strongly associated with metabolic syndrome, which is a combined expression of metabolic disorders including abdominal obesity [8]. In several studies, CRP levels already showed positive and significant correlations with body fat mass and VFA measured by CT [30, 31] in men. These results suggested that CRP levels can reflect inflammation by visceral adiposity. Previously published data demonstrated a highly significant association between smoke exposure and MDA [32, 33]; however, in the present study, the levels of urinary MDA in smokers were not elevated compared with nonsmokers.

This study has several limitations, which include a relatively small sample size and a study population limited to relatively healthy middle-aged men. However, the use of this population limits the effects of various unmeasured confounding factors. Unfortunately, we did not measure blood MDA concentration and could not evaluate their correlation with urinary MDA and VFA. In addition, the study was conducted using a cross-sectional design, and thus further studies are required. Nonetheless, we believe our findings are meaningful because this study represents a new attempt to evaluate multiple anthropometric parameters evaluating obesity including visceral abdominal fat area and various metabolic parameters. Furthermore, we suggest that urinary MDA levels may be useful as a marker of inflamed adipose tissue.

## 5. Conclusion

Urinary MDA, serum GGT, and serum CRP were significantly positively correlated with VFA, even after adjusting for BMI and WC in middle-aged healthy men. Further study is needed to confirm the validity of urinary MDA as a marker of inflamed adipose tissue.

## Figures and Tables

**Table 1:** General characteristics of subjects.

Characteristics	Value
Number of participants	73
Age (y; mean ± SD)	51.2 ± 5.8
Height (cm; mean ± SD)	171.4 ± 5.3
Weight (kg; mean ± SD)	71.8 ± 7.7
BMI (kg/m^2^; mean ± SD)	24.4 ± 2.1
Waist circumference (cm; mean ± SD)	86.3 ± 6.7
Abdominal VFA (cm^2^; mean ± SD)	100.8 ± 35.2
Abdominal SFA (cm^2^; mean ± SD)	127.6 ± 50.2

BMI: body mass index; VFA: visceral fat area; SFA: subcutaneous fat area.

**Table 2:** Characteristics of patients in the two study groups.

	Nonvisceral obesity (*n* = 38)	Visceral obesity (*n* = 35)	*P* value
Sociodemographic parameters			
Age (years)	50.3 ± 6.1	52.2 ± 5.5	0.185
Smoking status			0.162
Nonsmoker	24 (60.0)	16 (40.0)	
Smoker	14 (42.4)	19 (57.6)	
Alcohol consumer	30 (50.8)	33 (49.2)	0.770
Activity (METS/week)^*∗*^	1602.3 ± 2000.8	907.1 ± 932.2	0.268
Dietary parameters			
Energy intake (Kcal/kg/day)	28.9 ± 5.0	28.0 ± 5.1	0.475
Protein intake (g/kg/day)	1.2 ± 0.3	1.2 ± 0.3	0.800
Fat intake (g/kg/day)	0.7 ± 0.2	0.7 ± 0.2	0.543
Carbohydrate (g/kg/day)	4.6 ± 0.7	4.3 ± 0.8	0.163
Anthropometric parameters			
Body mass index (kg/m^2^)	23.8 ± 1.8	25.1 ± 2.2	**0.009**
Waist circumference (cm)	83.9 ± 5.1	88.8 ± 7.4	**0.002**
Sagittal abdominal diameter (cm)	18.3 ± 1.4	19.8 ± 2.6	**0.003**
DXA-measured fat			
Upper arm fat (%)	24.8 ± 4.6	26.4 ± 4.5	0.139
Lower leg fat (%)	23.6 ± 4.0	25.1 ± 4.6	0.148
Trunk fat (%)	27.6 ± 3.9	30.6 ± 5.1	**0.005**
Total fat (%)	25.6 ± 3.4	27.8 ± 4.2	**0.014**
CT-measured abdominal fat area			
VFA (cm^2^)^*∗*^	74.9 ± 16.4	128.8 ± 27.9	**<0.001**
SFA (cm^2^)^*∗*^	120.2 ± 42.1	135.7 ± 57.3	0.117
Metabolic parameters			
ALT (IU/L)^*∗*^	31.2 ± 17.0	35.1 ± 16.0	0.156
GGT (IU/L)^*∗*^	46.2 ± 28.7	99.5 ± 99.5	**0.008**
Uric acid (IU/L)^*∗*^	6.2 ± 1.0	7.1 ± 1.2	**0.002**
CRP (mg/dL)^*∗*^	0.10 ± 0.07	0.18 ± 0.25	0.147
LDL-cholesterol (mg/dL)	138.1 ± 35.6	137.9 ± 32.9	0.981
HDL-cholesterol (mg/dL)^*∗*^	53.2 ± 10.7	49.3 ± 10.9	0.154
TG (mg/dL)^*∗*^	132.4 ± 72.0	173.2 ± 97.0	0.063
Oxidative stress parameter			
Urinary MDA^*∗*^ (*µ*mol/g creatinine)	1.56 ± 0.85	2.08 ± 1.16	**0.018**

Data are expressed as the mean ± SE or the number (%).

*P* value by 2-sample *t*-test or chi-square test.

VFA: visceral fat area; SFA: subcutaneous fat area; DXA: dual-energy X-ray absorptiometry; ALT: alanine aminotransferase; GGT: *γ*-glutamyl transferase; CRP: C-reactive protein; LDL: low-density lipoprotein; HDL: high-density lipoprotein; TG: triglyceride; MDA: malondialdehyde.

One MET is roughly equivalent to 1 kcal/min for a person weighing 60 kg.

^*∗*^ *P* value by Mann-Whitney *U* test.

**Table 3:** Correlations between obesity indices, metabolic parameters, and urinary malondialdehyde levels.

	BMI	WC	Fat percent	VFA	SFA
ALT	0.394^**∗**^	0.305^**∗**^	**0.277** ^**∗**^	0.154	0.384^**∗**^
GGT	0.355^†^	0.335^†^	**0.380** ^†^	0.389^†^	0.376^†^
Uric acid	0.112	0.168	0.105	0.263^**∗**^	0.066
CRP	0.343^†^	0.346^†^	**0.290** ^**∗**^	0.326^†^	0.345^†^
TG	0.255^**∗**^	0.274^**∗**^	**0.257** ^**∗**^	0.254^**∗**^	0.233^**∗**^
Urinary MDA	0.081	0.031	−0.013	0.357^†^	0.005

ALT: alanine aminotransferase; GGT: *γ*-glutamyl transferase; CRP: C-reactive protein; TG: triglyceride; MDA: malondialdehyde; BMI: body mass index; VFA: visceral fat area; SFA: subcutaneous fat area; WC: waist circumference.

*P* value by Spearman's correlation.

^**∗**^ *P* < 0.05, ^†^ *P* < 0.01.

**Table 4:** Multiple regression analysis of associations between abdominal visceral fat area and metabolic parameters.

Abdominal VFA	Adjusted BMI				*P* value	Adjusted BMI and WC				*P* value
	*F*	*B*	SE	*β*		*F*	*B*	SE	*β*	
GGT	14.03^^†^^	0.106	0.047	0.230	0.028^**∗**^	10.70^†^	0.095	0.047	0.207	0.046^**∗**^
Uric acid	12.43^†^	5.703	3.240	0.185	0.083	10.23^†^	4.680	3.196	0.152	0.148
CRP	15.34^†^	53.703	20.231	0.278	0.010^**∗**^	11.296^†^	47.434	20.396	0.245	0.023^**∗**^
TG	11.17^†^	0.035	0.043	0.086	0.419	9.008^†^	0.028	0.042	0.069	0.507
Urinary MDA	19.37^†^	12.321	3.301	0.362	**<**0.001^†^	14.389^†^	11.867	3.259	0.349	0.001^†^

GGT: *γ*-glutamyl transferase; CRP: C-reactive protein; TG: triglyceride; MDA: malondialdehyde; BMI: body mass index; VFA: visceral fat area; WC: waist circumference.

^**∗**^ *P* < 0.05, ^†^ *P* < 0.01.
